# Case Report: triple therapy with pembrolizumab, ipilimumab, and lenvatinib in mitotane- and radiation-refractory, cortisol-secreting stage IV adrenocortical carcinoma: a genomically guided approach

**DOI:** 10.3389/fimmu.2026.1819473

**Published:** 2026-05-04

**Authors:** Afif Nakhleh, Ahmad Mahamid, Yaniv Yechiel, Leonard Saiegh, Sagit Zolotov, Salomon M. Stemmer

**Affiliations:** 1 Diabetes and Endocrinology Clinic, Maccabi Healthcare Services, Haifa, Israel; 2 Institute of Endocrinology, Diabetes and Metabolism, Rambam Health Care Campus, Haifa, Israel; 3 Azrieli Faculty of Medicine, Bar-Ilan University, Safed, Israel; 4 Rappaport Faculty of Medicine, Technion Israel Institute of Technology, Haifa, Israel; 5 Department of Surgery, Carmel Medical Center, Haifa, Israel; 6 Department of Nuclear Medicine, Rambam Health Care Campus, Haifa, Israel; 7 Institute of Endocrinology, Bnai Zion Medical Center, Haifa, Israel; 8 Davidoff Center, Rabin Medical Center, Petah Tikva, Israel; 9 Faculty of Medicine, Tel Aviv University, Tel Aviv, Israel

**Keywords:** adrenocortical carcinoma, immunotherapy, ipilimumab, lenvatinib, pembrolizumab

## Abstract

Adrenocortical carcinoma (ACC) is a rare malignancy with limited therapeutic options following the failure of standard adjuvant therapies. We present a 54-year-old female with stage IV cortisol-secreting ACC that progressed despite radical adrenalectomy, adjuvant radiation, and mitotane therapy. Comprehensive genomic profiling revealed a complex molecular landscape: a “double hit” driving hypercortisolism (*GNAS* activation and *HSD11B2* alteration) and a high tumor mutational burden (11.6 mutations/megabase) driven by converging instability mechanisms (*MSH6, TP53, DAXX, DNMT3A*, and *KMT2A* somatic mutations, and *TOP2A* overexpression), despite stable microsatellite status. Guided by this profile, a salvage regimen of pembrolizumab, ipilimumab and lenvatinib was initiated. The patient achieved a profound clinical response with resolution of Cushing’s syndrome and induction of adrenal insufficiency. Follow-up 18F-FDG PET/CT confirmed a sustained partial response per response evaluation criteria in solid tumors (RECIST) 1.1, accompanied by significant metabolic improvement with regression of metastatic burden with evident central photopenia in peritoneal lesions. This case illustrates how multi-faceted genomic analysis can rationalize aggressive combination immunotherapy in refractory ACC.

## 1 Introduction

Adrenocortical carcinoma (ACC) is a rare and aggressive endocrine malignancy with an incidence of 1–2 cases per million per year (1). The prognosis for metastatic disease is notoriously poor, with a 5-year survival rate of 10-20% (1). The current first-line therapies for advanced disease are mitotane monotherapy or the EDP-M regimen (etoposide, doxorubicin, and cisplatin plus mitotane) which yields objective response rates of less than 25% with significant toxicity (2).

The landscape of treatment is evolving with the advent of molecular profiling. While ACC has traditionally been considered an immunologically “cold” tumor, a subset of patients exhibits features that may predict response to immune checkpoint inhibitors (ICIs), including alterations in mismatch repair (MMR) proteins, high microsatellite instability (MSI), or high tumor mutational burden (TMB) (3). However, clinical efficacy with single-agent ICIs such as pembrolizumab, nivolumab, and avelumab has been mixed, generally yielding modest objective response rates between 6% and 23% due to the tumor’s typically low somatic mutation burden and immunosuppressive microenvironment (3, 4). While biomarkers like PD-L1 expression have shown limited predictive value, pembrolizumab has demonstrated durable benefit, supporting its inclusion in NCCN guideline for select cases (5–8).

To overcome resistance mechanisms such as cortisol-mediated immune suppression, emerging strategies are increasingly focusing on combination therapies, where dual checkpoint blockade or the addition of multi-kinase inhibitors (MKIs) has shown higher response rates in early trials compared to monotherapy (9, 10).

Here, we report the case of a patient with mitotane- and radiation-refractory, cortisol-secreting ACC who achieved a favorable sustained partial response, confirmed by both response evaluation criteria in solid tumors (RECIST) 1.1 criteria and metabolic PET assessment, to a salvage “triple therapy” regimen consisting of pembrolizumab, ipilimumab and lenvatinib. We utilized comprehensive next-generation sequencing (NGS) to dissect the complex molecular architecture of the tumor, identifying converging mechanisms of hormonal dysregulation (*GNAS, HSD11B2*), genomic instability (*MSH6, DAXX, TOP2A*), and mechanisms suggestive of immune resistance (*ZNRF3, AXIN1*) that provided the rationale for this precision approach.

## 2 Case presentation

### 2.1 Initial presentation and diagnosis

**Figure 1:**
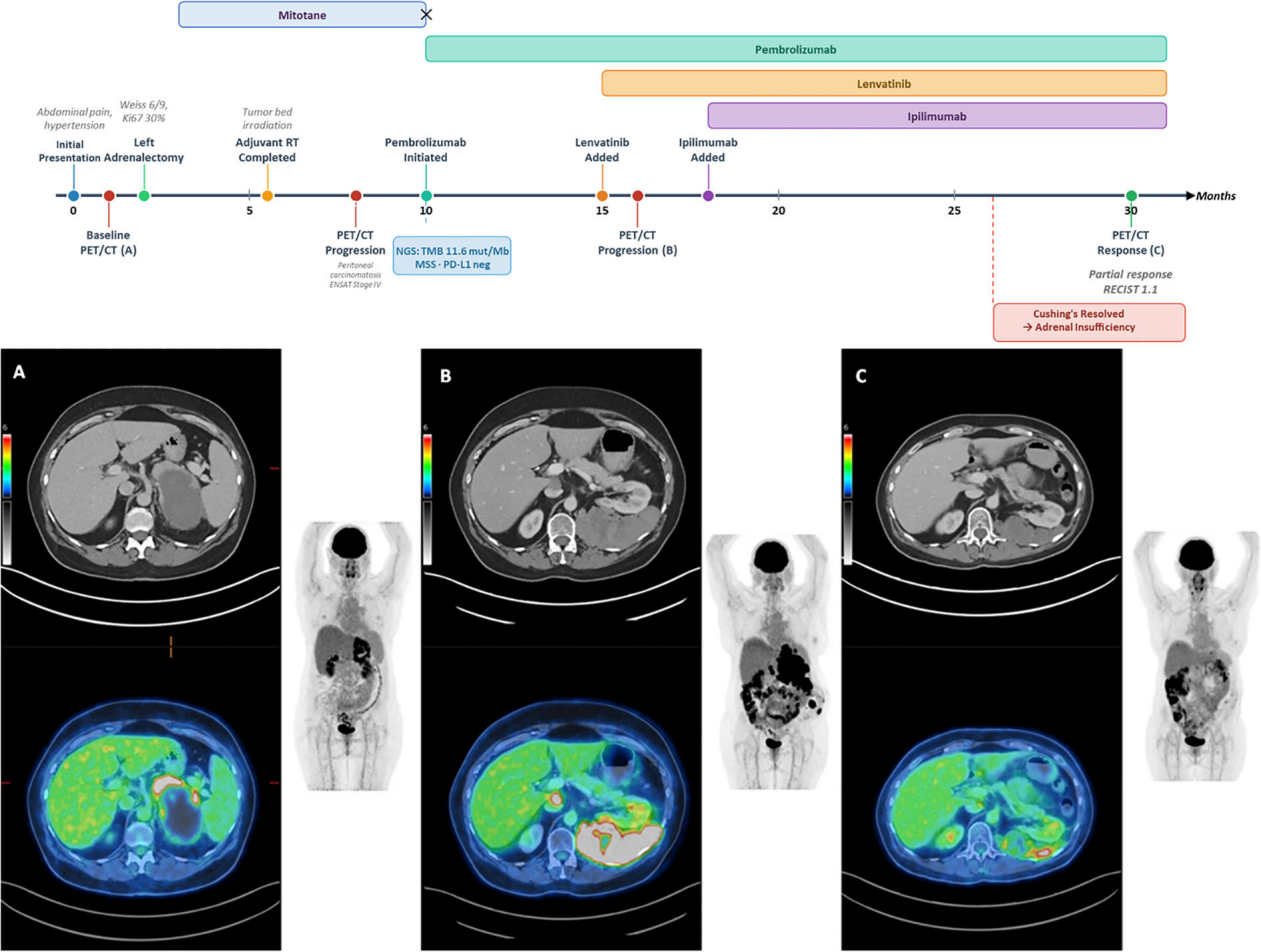
Clinical timeline and 18F-FDG PET/CT scans of ACC; representative maximum intensity projection (MIP), axial CT, and fused PET/CT images demonstrating disease staging **(A)** progression **(B)** and therapeutic response **(C)**. (upper panel) A clinical timeline summarizing the key diagnostic, surgical, and therapeutic milestones from initial presentation through the current follow-up. **(A)** baseline imaging reveals a large, heterogeneous left adrenal mass characterized by a central necrotic core and intense peripheral pathological uptake, consistent with primary adrenocortical carcinoma. **(B)** follow-up imaging demonstrates significant disease progression. Extensive FDG-avid lesions are noted within the adrenal bed, accompanied by widespread metastatic involvement including abdominal and pelvic lymphadenopathy, as well as peritoneal spread. **(C)** Post-treatment evaluation shows a favorable partial response per RECIST 1.1, with significant metabolic improvement. There is a notable reduction in tracer avidity and a partial regression of the local recurrence and the majority of previously identified metastatic lesions.

A 54-year-old woman presented approximately 2.5 years prior to the current evaluation with abdominal pain and new-onset hypertension. An initial abdominal computed tomography (CT) scan revealed a 10-cm heterogeneous mass in the left upper retroperitoneum, causing caudal displacement of the left kidney. Findings were suggestive of a hemorrhaging adrenal mass. A concomitant 18F-FDG PET/CT confirmed a large left adrenal mass (10 × 6.5 × 9 cm) with a necrotic hypodense core and pathologic peripheral FDG uptake (**Figure 1A**).

Clinical and biochemical evaluation revealed evidence of hypercortisolism and hyperandrogenism. Laboratory workup demonstrated elevated 24-hour urinary free cortisol (493 mcg/24h; upper limit of normal [ULN] 75, urine volume 1550 mL), suppressed adrenocorticotropic hormone (ACTH) (< 5 pg/mL), and elevated total testosterone (12.6 nmol/L). 24-hour urinary metanephrines (37 mcg/24h) and normetanephrines (203 mcg/24h) were normal (urine volume 2820 mL). Serum aldosterone (4.7 ng/dL) and renin (5.2 µIU/mL) levels were also within normal limits. The patient subsequently underwent an open left adrenalectomy. Histopathological examination confirmed ACC with a Weiss criteria score of 6/9 and a proliferation index (Ki67) of 30%.

Adjuvant therapy was initiated with mitotane (titrated to 3 g/daily). The patient also completed adjuvant radiation therapy to the tumor bed approximately three months post-operatively.

### 2.2 Disease progression

Despite multimodal adjuvant therapy, surveillance 18F-FDG PET/CT performed six months post-surgery demonstrated extensive hypermetabolic peritoneal carcinomatosis. Findings include multiple FDG-avid masses involving the gastrohepatic ligament, omentum, and the left abdominal musculature, consistent with ENSAT Stage IV metastatic adrenocortical carcinoma. Clinically, the patient manifested severe, debilitating Cushing’s syndrome (moon facies, proximal myopathy, hyperglycemia), confirming the functional recurrence of the tumor. Mitotane was discontinued due to tumor progression, and steroidogenesis inhibitors (ketoconazole, metyrapone) provided inadequate biochemical and clinical control and were subsequently discontinued.

### 2.3 Comprehensive genomic profiling

To identify actionable targets, DNA NGS was performed on the tumor specimen using the Tempus xT assay (Tempus Labs, Chicago, IL). The analysis revealed a tumor with a high TMB of 11.6 mutations/megabase (89th percentile), despite a microsatellite stable (MSS) status. PD-L1 expression was negative (tumor proportion score <1%).

Detailed analysis of somatic, biologically relevant, variants revealed a highly clonal tumor with loss of heterozygosity (LOH) across multiple driver genes, evidenced by variant allele fractions (VAF) exceeding 80%; the complete list of variants and their functional significance is provided in **Table 1**.

**Table 1:** Comprehensive genomic profiling (Tempus xT) revealing a highly clonal, multi-hit molecular landscape.

Biological domain	Gene	Variant (protein change)	Variant type	VAF (%)	Functional consequence and clinical relevance
Endocrine dysregulation	*GNAS*	p.R201H	Missense (GOF)	91.7%	Canonical activation of the Gs-α subunit; driver of constitutive cAMP signaling and autonomous hypercortisolism.
	*HSD11B2*	p.Y299H	Missense	85.6%	Impairment of cortisol-to-cortisone conversion, amplifying local cortisol excess.
	*CREBBP*	p.R1702C	Missense	86.0%	Alteration in CREB-binding protein; epigenetic deregulation of steroidogenic gene transcription.
Genomic instability	*MSH6*	p.R1172fs	Frameshift (LOF)	80.6%	Defect in MutSα mismatch repair complex; primary driver of TMB-H phenotype.
	*TP53*	p.R273C	Missense (LOF)	84.0%	Disruption of central DNA damage checkpoint; permits accumulation of somatic mutations.
	*DAXX*	p.G61fs	Frameshift (LOF)	83.2%	Chromatin remodeling defect linked to telomeric instability (ALT phenotype).
	*DNMT3A*	p.R736C	Missense (LOF)	85.0%	Epigenetic dysregulation of DNA methylation.
	*KMT2A*	p.R652*	Nonsense (LOF)	81.1%	Epigenetic dysregulation of histone methylation.
	*TOP2A*	Overexpression	N/A	N/A	Marker of extreme replication stress and high proliferative activity.
Wnt pathway and immune resistance	*ZNRF3*	p.R245*	Nonsense (LOF)	83.9%	Loss of a negative regulator of Wnt/β-catenin pathway; drives constitutive signaling and immune exclusion.
	*AXIN1*	p.G514R	Missense	83.4%	Impairment of β-catenin destruction complex; reinforcing Wnt activation.
Clonal markers of hypermutation	*EPHA7*	p.R762H	Missense	82.0%	Confirming high clonality (LOH) and hypermutated state.
	*CXCR4*	p.L41fs	Frameshift	81.9%	Confirming high clonality (LOH) and hypermutated state.
	*MYL1*	p.V7M	Missense	81.3%	Confirming high clonality (LOH) and hypermutated state.

ALT, alternative lengthening of telomeres; cAMP, cyclic adenosine monophosphate; CREB, cAMP response element-binding protein; DNA, deoxyribonucleic acid; GOF, gain of function; LOF, loss of function; LOH, loss of heterozygosity; N/A, not applicable; TMB-H, high tumor mutational burden; VAF, variant allele fraction.

These variants were categorized into three biologically relevant domains (**Table 1**): (i) endocrine dysregulation, driven by a “hormonal engine” comprising GNAS activation alongside HSD11B2 and CREBBP alterations; (ii) genomic instability, resulting from converging defects in mismatch repair, DNA damage checkpoints, chromatin remodeling, and epigenetic regulation; and (iii) a Wnt pathway activation profile suggestive of intrinsic immune resistance, via ZNRF3 and AXIN1 mutations.

### 2.4 Therapeutic rationale and salvage regimen

The genomic profile presented a unique therapeutic window. Although the tumor was MSS, the high TMB suggested a high neoantigen load potentially recognizable by the immune system. However, the presence of pathogenic *ZNRF3* and *AXIN1* mutations suggests constitutive Wnt pathway activation, a known driver of T-cell exclusion and resistance to single-agent PD-1 blockade (11).

Consequently, a combinatorial strategy was adopted to simultaneously enhance antigen presentation, reinvigorate T-cells, and modulate the suppressive microenvironment. The regimen was intensified sequentially:

1. Pembrolizumab (anti-PD-1): Initiated approximately 20 months prior to the current evaluation. The patient received 4 induction cycles of 400 mg IV every 6 weeks, followed by a maintenance dose of 200 mg IV every 3 weeks.
2. Lenvatinib (MKI): Added approximately 15 months prior to current evaluation (5 months after pembrolizumab initiation). Initially started at 10 mg daily PO, the dose was reduced to 4 mg daily due to weakness and mucocutaneous toxicity and subsequently re-titrated to 8 mg daily as tolerance improved.
3. Ipilimumab (anti-CTLA-4): Added 12 months prior to current evaluation. Administered at 80 mg IV every 3 weeks.

Importantly, a follow-up 18F-FDG PET/CT performed approximately six months after initiating pembrolizumab monotherapy (one month after initiating lenvatinib) and prior to the addition of ipilimumab (**Figure 1B**) demonstrated significant disease progression. Extensive FDG-avid lesions were noted within the adrenal bed, accompanied by widespread metastatic involvement, including abdominal and pelvic lymphadenopathy and peritoneal spread.

### 2.5 Clinical and radiological outcome

Approximately 12 months into the triple regimen, the patient’s clinical status underwent a significant transformation. The signs of severe Cushing’s syndrome completely resolved, replaced by symptoms and signs of adrenal insufficiency, including profound fatigue and hypotension. Notably, this state persisted despite the discontinuation of steroidogenesis inhibitors (ketoconazole and metyrapone). Laboratory evaluation confirmed primary hypoadrenalism: cortisol was low (34 nmol/L), adrenocorticotropic hormone was high (149 pg/mL), aldosterone low (2.3 ng/dL) with elevated renin (88.4 µIU/mL), and androgens were undetectable. Consequently, replacement therapy with hydrocortisone (10 mg twice daily) and fludrocortisone (0.05 mg daily) was initiated. Concurrently, the patient developed hypothyroidism, highly suspected to be an immune-related adverse event (irAE), which was managed with levothyroxine (500 mcg weekly). Importantly, this irAE did not necessitate the interruption or discontinuation of immune checkpoint inhibitor therapy.

Treatment tolerance was managed proactively. The patient experienced weakness and mucocutaneous toxicity (manifesting as grade 2 aphthous stomatitis), as well as arthralgia affecting the small joints of the hands. These adverse events led to the dose reduction of lenvatinib to 4 mg daily, before successful re-titration to 8 mg daily.

Serial 18F-FDG PET/CT imaging (**Figure 1C**) confirmed a favorable partial response per RECIST 1.1 criteria, accompanied by significant metabolic improvement on PET assessment. Comparison of the last two serial scans confirmed that the reduction in the sum of the longest diameters of the target lesions met the threshold for a RECIST 1.1 partial response (≥30% decrease from baseline). Concurrently, there was a marked reduction in FDG avidity across all measurable lesions. The dominant posterior upper abdominal mass demonstrated marked regression (reducing to 8.4 x 3.4 cm) with extensive central photopenia, indicative of cystic necrosis. Furthermore, the lateral abdominal wall nodule resolved completely. No new hypermetabolic lesions were identified, and the patient maintains an excellent performance status on maintenance therapy.

## 3 Discussion

This case highlights the power of integrating comprehensive genomic profiling with endocrine biology to guide salvage therapy in refractory ACC. The patient’s response was not serendipitous but grounded in specific molecular liabilities identified by the NGS assay.

The severity of the patient’s Cushing’s syndrome is explained by a “multi-hit” dysregulation of cortisol biology (**Table 1**). The truncal *GNAS* mutation (p.R201H) drives constitutive cAMP signaling and autonomous cortisol synthesis (12), while the *HSD11B2* variant impairs local cortisol-to-cortisone conversion, effectively amplifying the hormonal signal (13). Additionally, the CREBBP mutation suggests epigenetic deregulation of steroidogenic gene transcription, potentially perpetuating an open chromatin state at cAMP-responsive loci and preventing the downregulation of cortisol synthesis (14). Importantly, recent evidence highlights that tumor-derived cortisol itself constitutes a major immunosuppressive mechanism in ACC, directly impairing T-cell function and potentially reducing the efficacy of immune checkpoint inhibitors (15). This dual role of cortisol, as both a clinical manifestation and a barrier to immunotherapy, underscores the importance of controlling hypercortisolism in these patients.

This convergence of increased synthesis and impaired breakdown likely rendered the tumor refractory to standard steroidogenesis inhibitors, necessitating the cytotoxic destruction of the tumor itself to control the endocrinopathy. The development of adrenal insufficiency serves as a highly specific biomarker, confirming that the treatment successfully eradicated these specific functional clones.

A central paradox in this case was the finding of High TMB (11.6 mutations/megabase) in an MSS tumor. Our analysis identifies a specific “instability signature” driven by the loss of multiple genome guardians (**Table 1**).

While the *MSH6* frameshift alone is a potent driver of mutagenesis, it was compounded by the loss of *TP53* (preventing apoptosis of mutated cells) and *DAXX*. *DAXX* loss is a hallmark of ACC associated with the alternative lengthening of telomeres phenotype, which induces gross chromosomal instability (4).

Additionally, *DNMT3A* and *KMT2A* mutations confer epigenetic instability. Simultaneously, *TOP2A* overexpression, defective *TP53*, and high proliferation (Ki67 30%) drive extreme replication stress and error-prone synthesis. Additional high-VAF variants (*EPHA7, CXCR4, MYL1*) confirm a hypermutated state.

This resulted in a phenotype that generated sufficient neoantigens to prime an immune response, despite the absence of classic microsatellite instability. This underscores that MSS status alone should not preclude immunotherapy consideration in ACC; TMB must be evaluated independently. Indeed, high TMB has been proposed as a biomarker for dual checkpoint blockade efficacy in ACC (16).

The most critical barrier to immunotherapy in ACC is Wnt/β-catenin pathway activation, which excludes CD8+ T-cells from the tumor core (11). This patient harbored concurrent *ZNRF3* and *AXIN1* mutations (**Table 1**) suggesting that Wnt activation was a constitutive, obligate feature of the tumor.

In this context, single-agent pembrolizumab was predicted to have limited efficacy because T-cells could not penetrate the tumor stroma. The molecular architecture of this tumor justified the triple regimen as a synergistic, multi-step strategy.

Acting as a multi-kinase inhibitor (VEGFR1-3, FGFR1-4, PDGFRα, KIT, RET), lenvatinib critically modulates the immune landscape beyond simple anti-angiogenesis (9, 17). Through VEGFR-1 inhibition, it shifts tumor-associated macrophages from the pro-tumorigenic M2 phenotype to the anti-tumor M1 phenotype, facilitating T-cell recruitment. Simultaneously, lenvatinib-induced hypoxia upregulates PD-L1 expression, sensitizing the tumor to PD-1 blockade. Furthermore, by blocking VEGFR-2 on regulatory T cells, it mitigates their suppressive activity, effectively transforming the immune-excluded microenvironment into a permissible niche (17).

The combination of ipilimumab and pembrolizumab exploits non-redundant mechanisms to target distinct phases of the cancer-immunity cycle, exerting differential effects on CD4^+^ and CD8^+^ T cells (18). Ipilimumab (anti-CTLA-4) acts centrally within draining lymph nodes during the priming phase. As established by recent mechanistic studies, CTLA-4 targeting primarily affects T-cell priming and expands T-cell clonal diversity, preferentially driving the expansion of CD4+ effector T cells while dampening regulatory T cells (18–20). This repertoire broadening is critical in this patient to target the heterogeneous neoantigen landscape generated by MSH6/DAXX-driven instability. Complementing this, pembrolizumab (anti-PD-1) acts peripherally within the tumor microenvironment during the effector phase, where it specifically reinvigorates exhausted CD8+ T cells (20, 21). Thus, the combination achieves a synergistic effect: ipilimumab recruits a diverse polyclonal army against the high mutational burden, while pembrolizumab restores the cytotoxic lethality of these effectors at the tumor site.

It is important to acknowledge several limitations of this report. As a single case, the generalizability of these findings is limited, and the observed response cannot establish a causal relationship between the genomic profile and therapeutic efficacy. Furthermore, the sequential addition of agents makes it difficult to attribute the response to any single component. Prospective studies are needed to validate whether similar genomic profiles can reliably predict benefit from this combination in ACC.

## 4 Conclusion

In the era of targeted personalized medicine, an effort should be made to treat pre-curable diseases. This report provides a comprehensive molecular and clinical characterization of a patient with refractory ACC who achieved an exceptional response to triple therapy. The integration of genomic data clarified the tumor’s functional nature, resolved the mechanism of its immunogenicity, and rationalized the need for combinatorial therapy. This report supports the routine use of broad-panel NGS in advanced ACC to identify rare, actionable subsets of patients who may benefit from aggressive immunotherapy combinations. However, given the inherent limitations of a single-case observation, prospective validation in larger cohorts is warranted.

## Data Availability

The original contributions presented in the study are included in the article/supplementary material. Further inquiries can be directed to the corresponding author.
